# PET/CT Based *In Vivo* Evaluation of ^64^Cu Labelled Nanodiscs in Tumor Bearing Mice

**DOI:** 10.1371/journal.pone.0129310

**Published:** 2015-07-01

**Authors:** Pie Huda, Tina Binderup, Martin Cramer Pedersen, Søren Roi Midtgaard, Dennis Ringkjøbing Elema, Andreas Kjær, Mikael Jensen, Lise Arleth

**Affiliations:** 1 Structural Biophysics, Niels Bohr Institute, University of Copenhagen, Universitetsparken 5, DK-2100 København Ø, Denmark; 2 Cluster for Molecular Imaging and Department of Clinical Physiology, Nuclear Medicine & PET, Rigshospitalet and University of Copenhagen, Blegdamsvej 3, DK-2200 København N, Denmark; 3 Hevesy Laboratory, Center for Nuclear Technology, Technical University of Denmark, Frederiksborgvej 399, DK-4000 Roskilde, Denmark; Baker IDI Heart and Diabetes Institute, AUSTRALIA

## Abstract

^64^Cu radiolabelled nanodiscs based on the 11 α-helix MSP1E3D1 protein and 1-palmitoyl-2-oleoyl-sn-glycero-3-phosphatidylcholine lipids were, for the first time, followed *in vivo* by positron emission tomography for evaluating the biodistribution of nanodiscs. A cancer tumor bearing mouse model was used for the investigations, and it was found that the approximately 13 nm nanodiscs, due to their size, permeate deeply into cancer tissue. This makes them promising candidates for both drug delivery purposes and as advanced imaging agents. For the radiolabelling, a simple approach for ^64^Cu radiolabelling of proteins via a chelating agent, DOTA, was developed. The reaction was performed at sufficiently mild conditions to be compatible with labelling of the protein part of a lipid-protein particle while fully conserving the particle structure including the amphipathic protein fold.

## Introduction

Nanoparticle-based drug delivery systems are widely investigated with the aim of improving the pharmacological properties of conventional drugs and to solve problems with low solubility and poor biodistribution [1]. A central focus area lies in cancer treatment and diagnosis, where the ability of nanoparticles to accumulate in tumor tissues through the enhanced permeability and retention (EPR) effect is pursued [2, 3]. In this context, nanoparticles are used as drug delivery vehicles as well as imaging agents in cancer diagnosis and treatment selection, providing direct visualization of the general biodistribution and degree of tumor accumulation of nanoparticle-based anti-cancer drugs [4, 5].

Nanodiscs, engineered from human high density lipoprotein, are disc-shaped phospholipid bilayers stabilized by two surrounding α-helical amphipathic membrane scaffold proteins (MSPs). The MSPs keep the nanodiscs in their well-defined shape and size over a wide range of conditions including concentrations and temperatures [6–8]. The nanodisc system has been designed and optimized to be a platform for studying membrane proteins [9, 10]. In these applications, the phospholipid bilayer offers a perfect environment for membrane proteins and stabilize their natural conformation and function in a more native like state than more commonly used detergent-based systems [11]. Nanodiscs have shown promising results for drug delivery usage in for example the delivery of an influenza vaccine, as studies have shown them able to trigger a broad protective immune response in mice [12] and for a specific antigen which has shown a therapeutic potential regarding the autoimmune disease myasthenia gravis [13]. However, the full potential of the nanodiscs in this type of applications has not yet been elucidated as information regarding their biodistribution is sparse and still incomplete [14]. The present article presents, for the first time, a combined positron emission tomography (PET) and computed tomography (CT) real-time, non-invasive visualization of the nanodisc biodistribution *in vivo* including visualization of its permeation into cancer tumor tissue in a tumor bearing mouse model.

**Fig 1:**
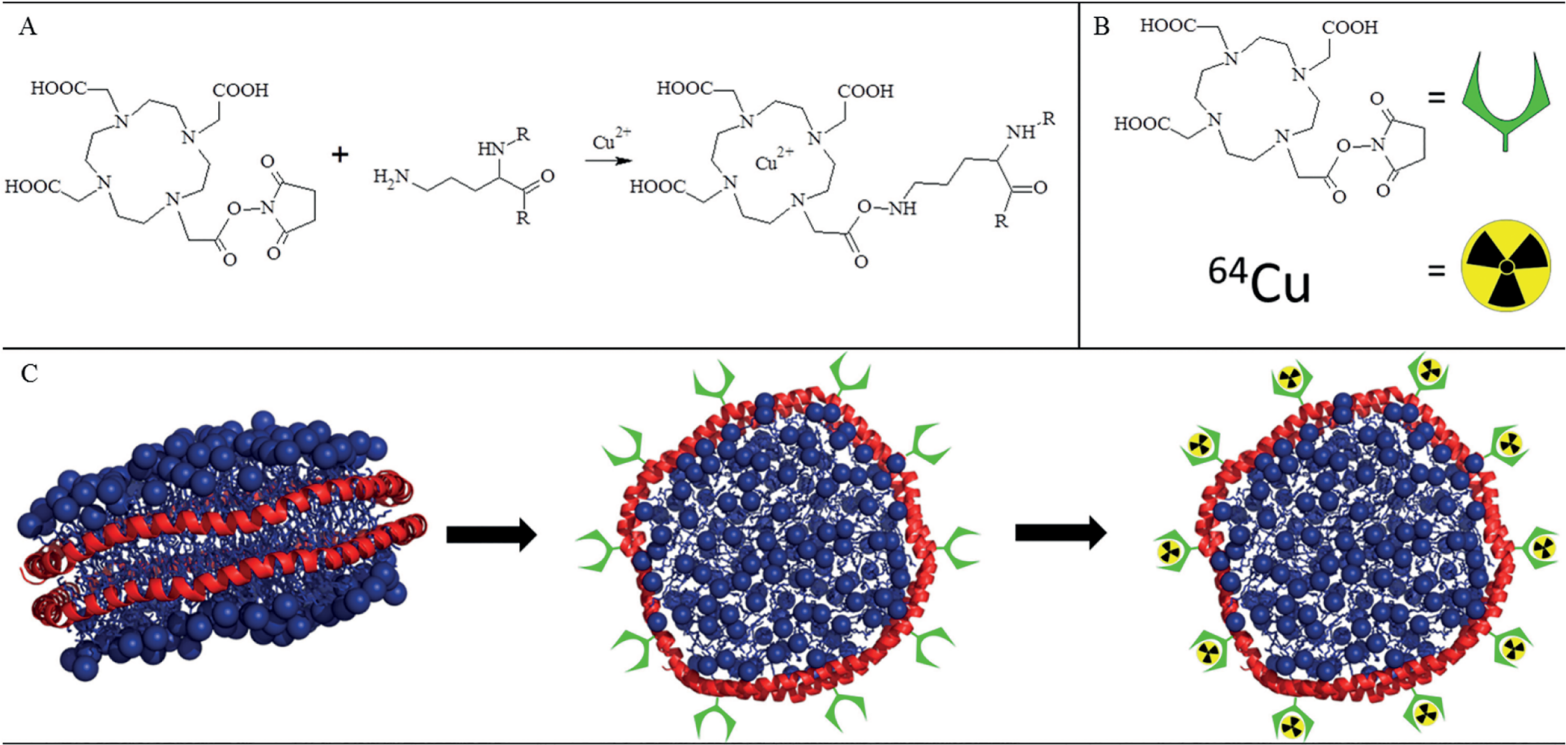
Schematics of nanodisc radiolabelling. A: chemical reaction of DOTA-NHS ester-conjugation to MSP lysines and further radiolabelling with ^64^Cu^2+^. B: symbol explanation for C. C 1: MD simulation of ordinary nanodisc[6] (lipids in blue, MSP in red). 2: nanodisc assembled with DOTA conjugated MSP. 3: ^64^Cu radiolabelled nanodisc.

To reach this goal, a simple method for ^64^Cu radiolabelling was developed allowing for conjugating the protein component of the nanodisc with the chelating agent 1,4,7,10-tetraazacyclododecane-1,4,7,10-tetraacetic acid (DOTA). This enabled binding of the radioactive isotope ^64^Cu (Fig 1) while at the same time involving minimal modifications of the biomolecule in order to avoid significant structural changes. An alternative DOTA-based approach has recently been proposed for the labeling of the 12 amino acid QFP peptide [15]. For the present work, we have developed a milder reaction approach that is compatible with proteins in solution and allow for labeling of the MSP, while conserving their amphipathic fold and ability to form nanodiscs. Therefore, the method is applicable to other protein systems. ^64^Cu was chosen as the radionuclide because of its relatively long half-life (12.7 hours) and relatively low maximum positron energy (0.66 MeV), which makes it very suitable for PET imaging. Nanodiscs were assembled with DOTA-conjugated MSPs and structurally characterized by combined small-angle x-ray scattering (SAXS) and small angle neutron scattering (SANS) confirming that the nanodisc structure indeed remained fully intact after DOTA-conjugation. The DOTA-conjugated nanodiscs were radioactively labeled with ^64^Cu and their *in vitro* stability evaluated in mouse blood plasma, showing that the ^64^Cu-labeled nanodiscs were sufficiently stable over time to be relevant for *in vivo* studies. Finally, ^64^Cu-labelled nanodiscs were intravenously administered to 10 tumor bearing nude mice and the biodistribution analyzed by PET and gamma counting of tissues of interest. This showed that the nanodiscs accumulate intensively in the tumor tissue as well as in the kidneys.

## Materials and Methods

### Materials

DOTA-NHS-ester was purchased from Macrocyclic, Dallas, Texas, USA. POPC was purchased from Avanti Polar Lipids, Alabaster, Alabama, USA. Sodium Cholate, Sodium Phosphate, NaCl and Dulbecco’s PBS were purchased from Sigma-Aldrich, St. Louis, Missouri, USA. Ammonium acetate was purchased from Merck, Damstadt, Germany. ^64^CuCl_2_ was obtained from the Hevesy Laboratory Risø DTU, Roskilde, Denmark.

### DOTA-conjugation of MSP

MSP1E3D1(-) (MSP) was expressed and purified as previously described [16]. 1, 3 or 5 molar equivalents 1,4,7,10-Tetraazacyclododecane-1,4,7,10-tetraacetic acid mono (N-Hydroxysuccinimide ester) (DOTA) to number of lysines from MSP were dissolved in sodium phosphate buffer (0.1 M NaPO_4_, 0.1 M NaCl, pH 8) containing MSP1E3D1(-) (4.7 mg/ml) and left for continuous stirring for 1 hour at 4°C. DOTA-conjugation was confirmed and analysed by MALDI TOF on a Microflex (Bruker, Billerica, MA, USA) and a Q-Tof Ultimate (Micromass, Beverly, MA, USA) using a HCCA matrix (Bruker, Billerica, MA, USA).

### Assembly and purification of nanodiscs

1-palmitoyl-2-oleoyl-sn-glycero-3-phosphatidylcholine (POPC) (20 mg/ml) in a chloroform stock solution was transferred to a glass vial. The chloroform was evaporated using a flow of nitrogen gas for 1 hour. Excessive chloroform was removed using a rotary evaporator overnight. Dry POPC was dissolved in cholate buffer (0.1 M NaPO_4_, 0.1 M NaCl, 20 mM sodium cholate, pH 8) to a concentration of 0.05 M. MSP-DOTA in a sodium phosphate buffer (0.1 M NaPO_4_, 0.1 M NaCl, pH 8) was mixed with POPC solubilized in cholate-containing buffer (0.1 M NaPO_4_, 0.1 M NaCl, 20 mM sodium cholate, pH 8) in a molar ratio of 130:1 POPC:MSP and left for continuous shaking for 90 min at 4°C. 0.5 ml Biobeads (BioRad, Hercules, CA, USA) to 1 ml sample was added to the mixture, which was left for continuous shaking over night at 4°C. The Biobeads were removed by centrifugation for 1 min. at 4000 RPM and filtration through a 0.22 μm syringe filter. DOTA-conjugated nanodiscs were purified by size exclusion chromatography (SEC) using a Superdex 200 column (10/300) (GE healthcare, Little Chalfont, UK) with a 0.5 ml/min flow using ammonium acetate buffer (0.1 M CH_3_CO_2_NH_4_, 0.1 M NaCl, pH 7.5) as eluent.

### ^64^Cu labelling and purification

1 ml nanodisc solution (approximately 12.8 mg/ml DOTA conjugated nanodiscs) in ammonium acetate buffer (0.1 M CH_3_CO_2_NH_4_, 0.1 M NaCl, pH 7.5) was added to a dry vial containing 587.2 MBq ^64^CuCl_2_ and reacted for 45 min at room temperature. The 1 ml solution was purified and analyzed by SEC using a Superdex 200 column (10/300) (GE healthcare, Little Chalfont, UK) with phosphate buffer saline (PBS) as eluent. A flow rate of 0.5 ml/min was applied and, ^64^Cu labelled nanodiscs were detected by UV280 nm and radioactivity by gamma detection using a NaI gamma detector (Bicron, Canaan, CT, USA).

### Structural characterization by SAXS and SANS

SAXS data were obtained at the BioSAXS instrument formerly at beamline ID14-3 at European Synchrotron Radiation Facility (ESRF) in Grenoble[17]. SANS data were obtained at the D11 SANS instrument at ILL[18]. For small-angle X-ray scattering, 50 μl samples of DOTA-conjugated (5.3 mg/ml) and unconjugated nanodiscs (4.2 mg/ml) in Tris buffer (20 mM Tris, 0.1 M NaCl, pH 7.5) and the Tris buffer backgrounds were measured at 20°C by slow flow through the X-ray beam in a capillary cuvette. The obtained data were azimuthally averaged and buffer background measurements were subtracted from sample measurements. The data were converted to absolute scale (i.e. intensities in units of cm/cm^3^) using BSA as a secondary standard. The data analysis was carried out on these background subtracted, absolute scaled scattering intensity data, *I(q)*. Prior to the small-angle neutron scattering experiments, the buffers of the two samples were exchanged to a D_2_O based Tris buffer (20 mM Tris, 0.1 M NaCl, pH 7.5) using centrifugation spin filters. The SANS measurements were performed in 2mm rectangular Hellma Quartz cuvettes at 20°C. A neutron wavelength of 6Å and sample to detector distances of, respectively, 1.5 m and 10.5 m were used in order to cover the desired *q*-range. Samples were exposed for 6 minutes at the 1.5 m setting and 15 minutes at the 10.5 m setting. Azimuthally averaged, background subtracted data were absolute scale calibrated using H_2_O as a secondary standard. Further data analysis was carried out on these background subtracted, absolute scaled scattering intensity data, *I(q)*.

### *In vitro* stability

^64^Cu-nanodiscs were prepared as described earlier. 0.5 mg ^64^Cu-nanodiscs in solution (2.75 mg/ml) with an activity of 6.2 MBq were incubated at 37°C in 1 ml 0.22μl filtered mouse plasma harvested from BomTac:NMRI-*Foxn1* ^*nu*^ nude mice and analyzed after 1, 12, 24 and 48 hours of incubation. The nanodiscs in plasma were analyzed by SEC using a BioSep-SEC-s2000 column with PBS buffer as eluent. A flow rate of 0.5 ml/min was applied. Eluate was detected by gamma detection using a NaI gamma detector (Bicron, Canaan, CT, USA).

### Animal models

Human lung carcinoid (NCI-H727) tumor cells (~10^7^), purchased from European Collection of Cell Cultures (ECACC, Porton Down, UK) were inoculated in the left and right flank of BomTac:NMRI-*Foxn1* ^*nu*^ nude mice (n = 10) in a 200 μl volume with a 1:1 mixture of suspended cells and Matrigel (BD Biosciences, San Jose, CA, USA). Tumors were allowed to grow for three weeks before scanning to ensure exponential tumor growth. Mice were fed with 1314 TPF (Altromin, Lage, Germany) and water ad libitum. All experiments involving animals were approved by the national authority. A computer tomography (CT) scan was acquired prior to the PET scan for anatomical localization of foci on a MicroCAT II system (Siemens Medical Solutions, Malvern, PA, USA). CT settings were a tube voltage of 61 kVp, a tube current of 500 μA, 360 rotation steps, an exposure time of 440 ms and a voxel size of 0.092 mm.

### Micro-PET/CT imaging

The mice were anaesthetized using 4% sevofluran (Abbott Scandinavia AB, Solna, Sweden) mixed with 35% O_2_ in N_2_ before injected intravenously with 10 MBq of ^64^Cu-labeled nanodiscs in a lateral tail vein. General anaesthesia was maintained using 2.5% sevofluran (Abbott Scandinavia AB, Solna, Sweden) mixed with 35% O_2_ in N_2_. Static PET scans were acquired, 1, 12, 24 and 48 hours post-injection on a dedicated small animal PET scanner (MicroPET Focus 120, Siemens Medical Solutions, Malvern, PA, USA). Acquisition times were 10, 15, 20 and 30 minutes for the 1, 12, 24 and 48 hour scans, respectively, to ensure proper signal-to-noise ratios. The voxel size was 0.866 x 0.866 x 0.796 mm^3^, and the resolution was 1.4 mm in the centre-field-of-view. Data were reconstructed with the maximum a posterior (MAP) reconstruction algorithm.

All animal care and experimental procedures were approved by the Danish Animal Welfare Council (Permit number: 2013–15–2934–00064).

### Quantitative analysis of PET images

PET and CT images were analysed as fused images using the Inveon software (Siemens). Regions of interest (ROIs) were drawn around liver, kidney, spleen, muscle, tumors and the left-ventricle. Uptake in the left ventricle of the heart was taking as a measure of the blood concentration. Percentage of injected dose per gram (%ID/g) was calculated.

### Gamma counting

Following the 48 hour PET scan, the mice were killed, blood collected and organs of interest harvested for endpoint measurements in a Wizard2 gamma-counter (Perkin Elmer, Skovlunde, Denmark) with a counting efficiency of 9.43% for ^64^Cu. Percentage injected dose per gram (%ID/g) and tumor-to-muscle ratios were calculated.

### Statistical analysis

Student t-test was applied for two-group comparisons and comparison of three or more groups was done using One-way ANOVA with Bonferroni-corrected post hoc test. PET and gamma counting results are reported as mean ±.SEM. Statistical analysis was performed using GraphPad Prism 5 software (GraphPad Software, Inc.). A p-value of 0.05 was considered significant.

## Results and Discussion

### DOTA-conjugation of MSP

The metal chelating reagent DOTA-NHS-ester (DOTA) was reacted onto lysine groups of the MSP, which was confirmed by MALDI TOF analysis (**Fig A** in S1 File). The reaction was carried out at physiological conditions in order to conserve the MSP. DOTA was chosen as the chelate, as it has been widely used in radiopharmaceuticals and is well characterized throughout the literature [19]. DOTA reacted satisfactory with MSP, and a molar ratio of 5:1 DOTA:lysines was found to be a suitable reaction condition, which resulted in a rough average of 5 DOTA bound to 1 MSP. This was believed to be an appropriate amount of chelate for the Cu-64 reaction, while at the same time it was not expected to affect the protein properties of MSP nor the nanodisc reconstitution.

### Assembly, size and structure of DOTA-conjugated nanodiscs

**Fig 2:**
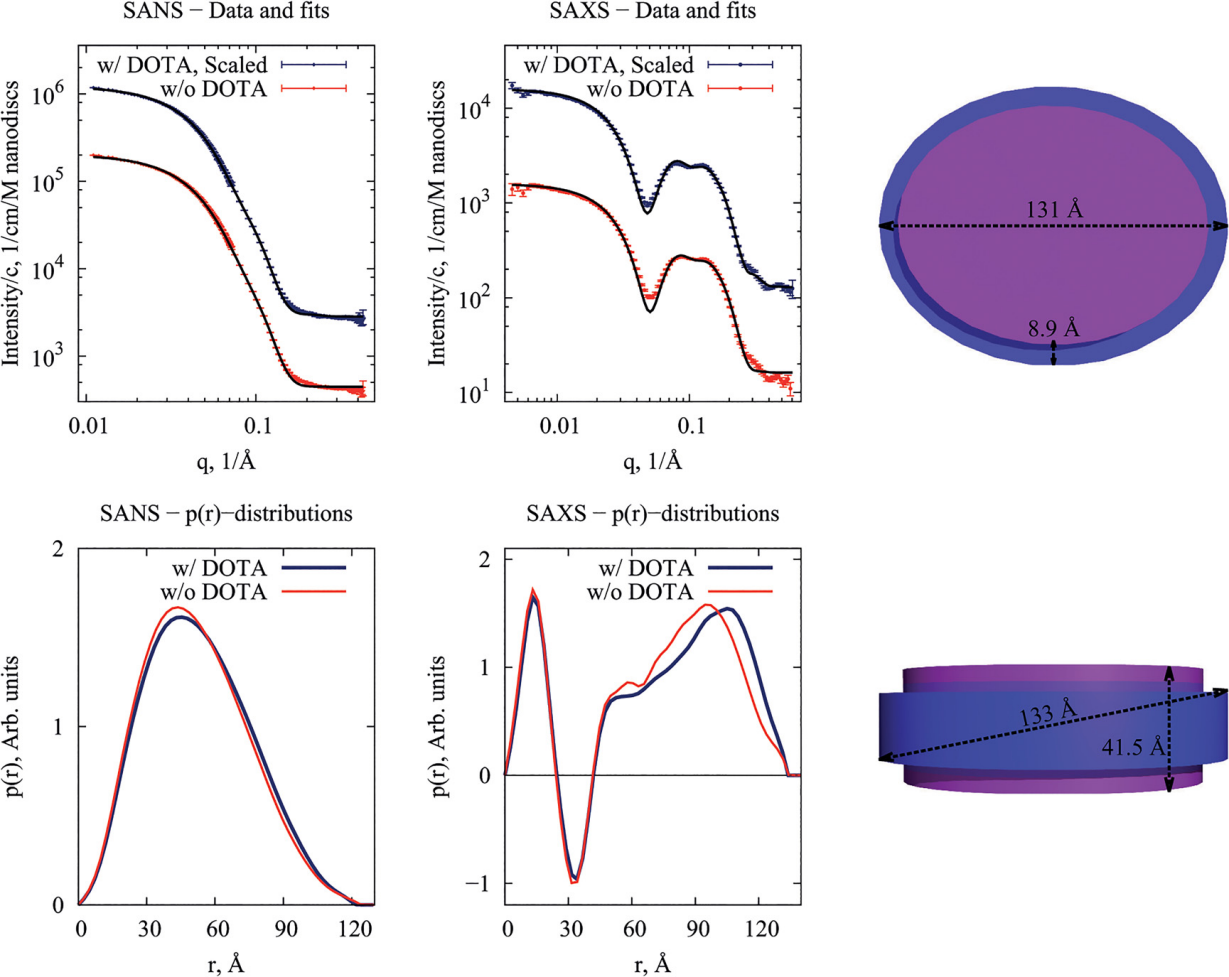
SAXS and SANS data of unmodified and DOTA conjugated nanodiscs. Top left plot: SANS data of unmodified nanodiscs (in red) and DOT- conjungated nanodiscs (in blue) with their respective fits. Top right plot: SAXS data of unmodified nanodiscs (in red) and DOTA-conjugated nanodiscs (in blue) with their respective fits. Bottom left plot: Pair-distance distribution functions, *p(r)*, refined from the obtained SANS data of unmodified nanodiscs (in red) and DOTA-conjugated nanodiscs (in blue). Bottom right plot: *p(r)*-distributions refined from the obtained SAXS data of unmodified nanodiscs (in red) and DOTA-conjugated nanodiscs (in blue). To the right, a rendering of the refined structure is shown.

The nanodiscs were assembled with DOTA-conjugated MSP and POPC before radiolabelling with ^64^Cu. DOTA-modified nanodiscs exhibited the same reconstitution behavior as unmodified nanodiscs as judged from size exclusion chromatography (SEC) data from the purification process, and in both cases evidence of well-defined discs was observed (**Fig B** in S1 File). SEC is a low-resolution technique and in order to obtain higher resolution structural evidence that the DOTA-conjugated nanodiscs had the same structure as the unmodified nanodiscs, a small-angle scattering study was performed. The obtained scattering data and the derived pair-distance distribution functions, *p(r)*, from DOTA conjugated and ordinary nanodiscs (Fig 2) were very similar and in accordance with previous observations from a smaller version of the discs [20]. The *p(r)*-functions show that unmodified and DOTA-nanodiscs have a diameter of approximately 130 Å. However, a small difference between the two is seen in the interval from 60 to 130 Å where the *p(r)*-functions of DOTA-modified nanodiscs are shifted towards larger distances. A model accounting for a slight anisotropy in the shape of the discs was generalized from a previous study [21] and fitted to the experimental data (see model and fits in Fig 2). In accordance with previous findings, each nanodisc was stabilized by two MSPs. From the fits, the following parameters describing the scattering from the unlabeled discs were obtained: The membrane scaffolding protein belt was refined to a thickness of 9.8 Å, the area per lipid headgroup was refined to 70.3 Å^2^, which is slightly above previously reported value of 62.7 Å^2^ observed in large bilayer membranes [22]; the partial specific molecular volume of MSP was inferred to be 37900 Å^3^ and similarly, the partial specific molecular volume of POPC was estimated to be 1280 Å^3^. From these parameters, one can derive that the number-weighted average number of POPC molecules per disc to be 188. For the DOTA-conjugated discs, we obtained a value of 8.9 Å for the thickness of the membrane scaffolding protein belt and a value of 61.6 Å^2^ for the area per lipid headgroup. The partial specific molecular volumes of MSP and POPC were refined to very similar values as for the unlabeled discs: 36800 Å^3^ and 1280 Å^3^, respectively. These parameters result in a number-weighted average number of lipids per disc of 247. Thus, from the analysis of the small-angle scattering data, we conclude that the overall disc-shape is fairly unaffected by the DOTA-conjugation. However, the conjugation does seem to introduce minor perturbations in the fine-structure of the particles.

### ^64^Cu-labelling of nanodiscs

**Fig 3:**
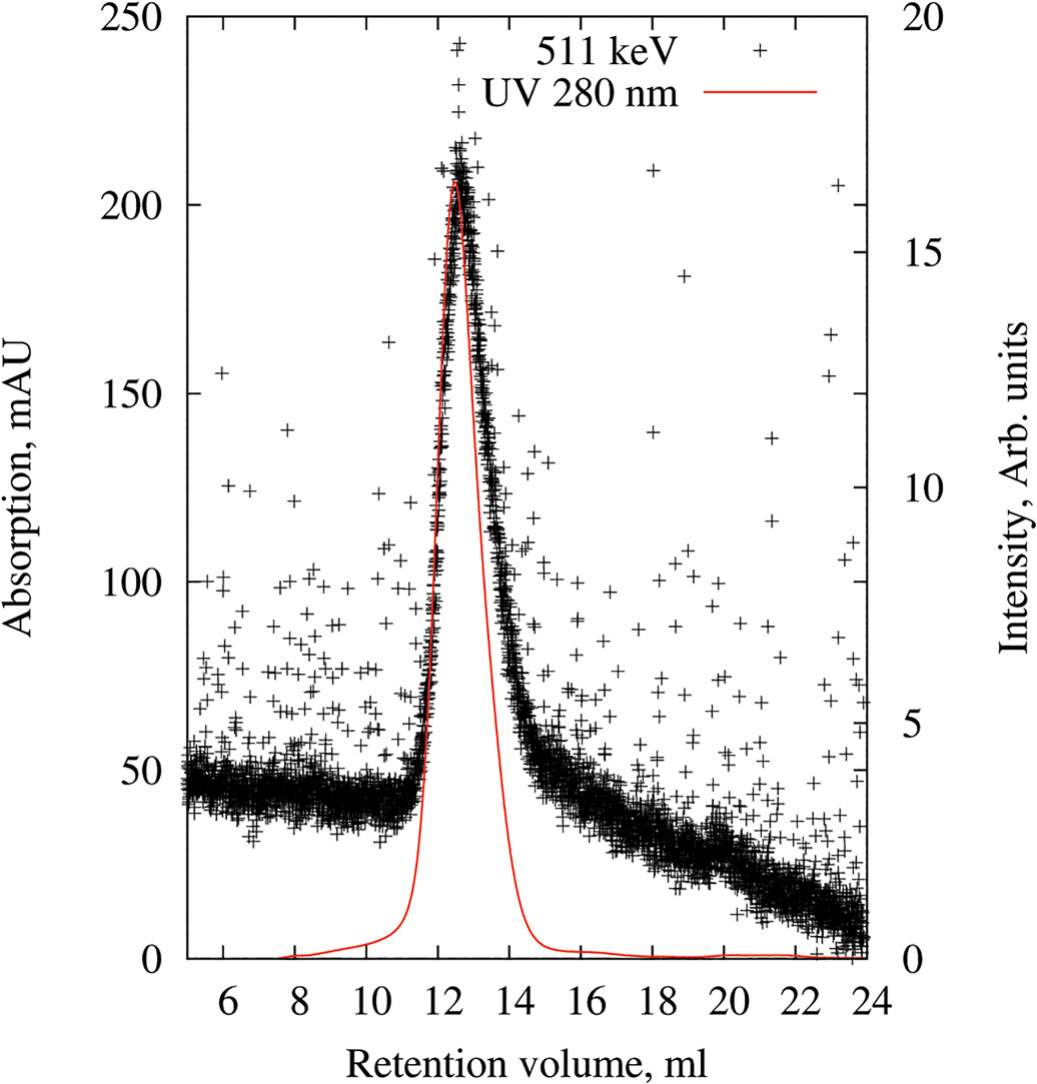
Purification of ^64^Cu-nanodiscs. Chromatogram obtained by size exclusion chromatography using a Superdex 200 (10/300) column detected by UV 280 nm absorption (mAU) and radioactivity at 511 kEV (intensity). ^64^Cu-nanodiscs were collected from the column at an elution time of 12.4 ml.

DOTA-modified nanodiscs were ^64^Cu-labelled by simple incubation with ^64^CuCl_2_ in a pH 7.5 ammonium acetate buffer at room temperature. The labelling was confirmed by SEC with dual detection of UV 280 nm and gamma-emission, which clearly showed that ^64^Cu was bound to the nanodisc fraction (Fig 3). The finding of efficient radiolabelling at these mild conditions was very satisfactory, as previous work reports the requirement for more harsh reaction conditions at lower pH [23–26], which is generally not compatible with the conservation of the structure and function of protein-based biomolecules.

**Fig 4:**
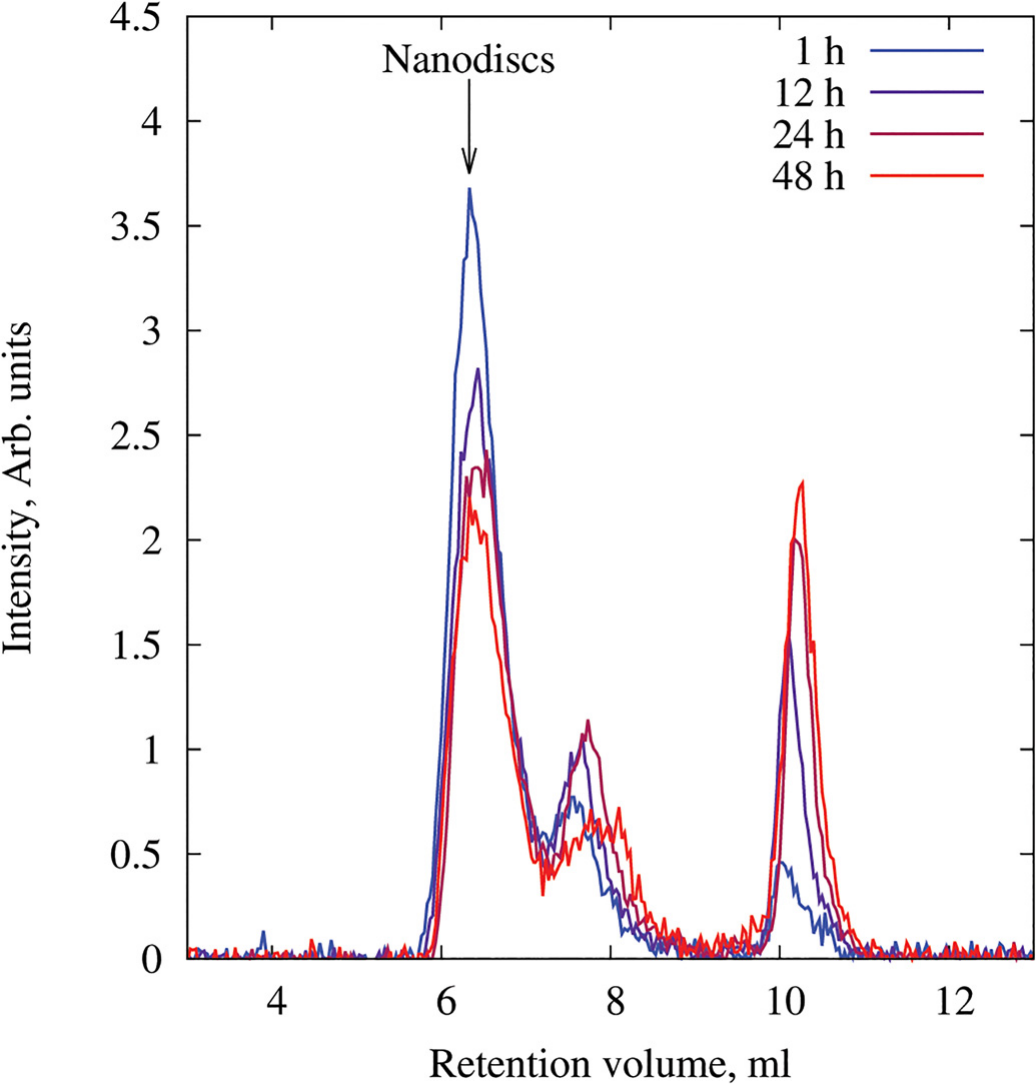
*In vitro* stability of ^64^Cu-nanodiscs in mouse plasma at 37°C. Chromatograms were obtained by size exclusion chromatography (SEC) using a BioSep-SEC-s2000 column and radioactivity detected at 511 kEV. ^64^Cu-nanodiscs eluted from the column at approximately 6.4 ml. Analyses were carried out after 1, 12, 24 and 48 hours of incubation. Data are background subtracted and normalized by the total counts.

The *in vitro* labelling stability of the nanodiscs was investigated prior to the *in vivo* biodistribution studies to examine to which extent an observed distribution could be assigned to nanodiscs and not only to degradation products or unbound CuCl_2_. For this study, the nanodiscs were incubated in mouse plasma at 37°C for 48 hours, and aliquots were evaluated by SEC after 1, 12, 24 and 48 hours. This indicated an overall good stability of the ^64^Cu-nanodiscs in mouse plasma at body temperature. After 48 hours of incubation, a dominating peak matching the retention time of nanodiscs could still be detected as seen in Fig 4. However, degradation products were observed already after 1 hour of incubation and increased over time when compared to the nanodisc fraction. It was not possible to determine if these smaller ^64^Cu-containing particles were degradation products or a result of transchelation between competing plasma proteins. The *in vitro* stability was judged sufficiently well for continuation of *in vivo* studies. However, a further improved radiolabelling stability may be obtained by using a chelate indicating higher *in vivo* stability and stronger affinity for Cu(II) than that of DOTA [27].

### PET/CT-based in vivo evaluation of ^64^Cu labelled nanodiscs in tumor bearing mice

**Fig 5:**
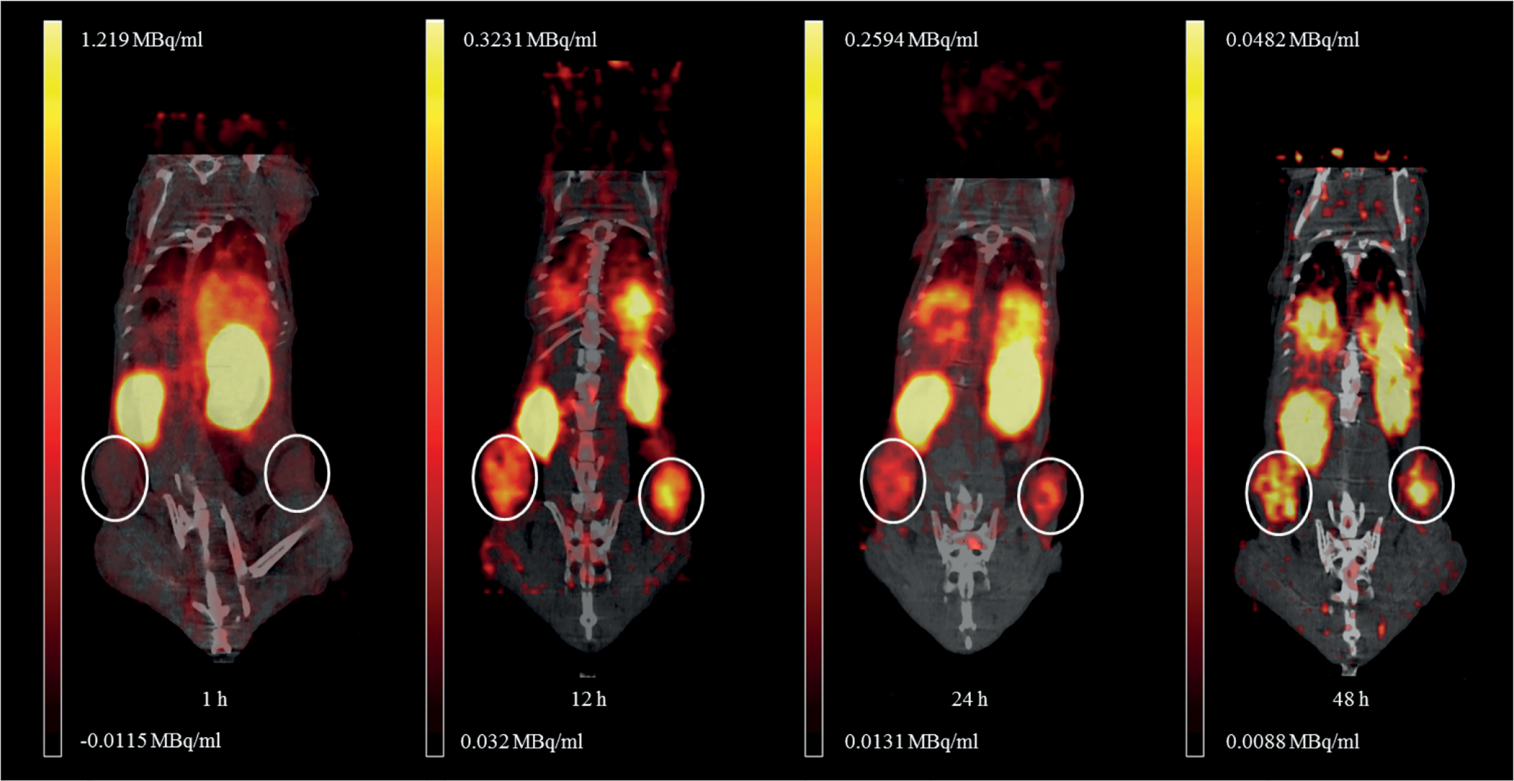
Positron emission tomography (PET)/computed tomography (CT) images of ^64^Cu-nanodisc distribution in tumor bearing nude mice. The images show the coronal plane of the mouse recorded 1, 12, 24 and 48 hours post injection. The tumors are circled in white.

**Fig 6:**
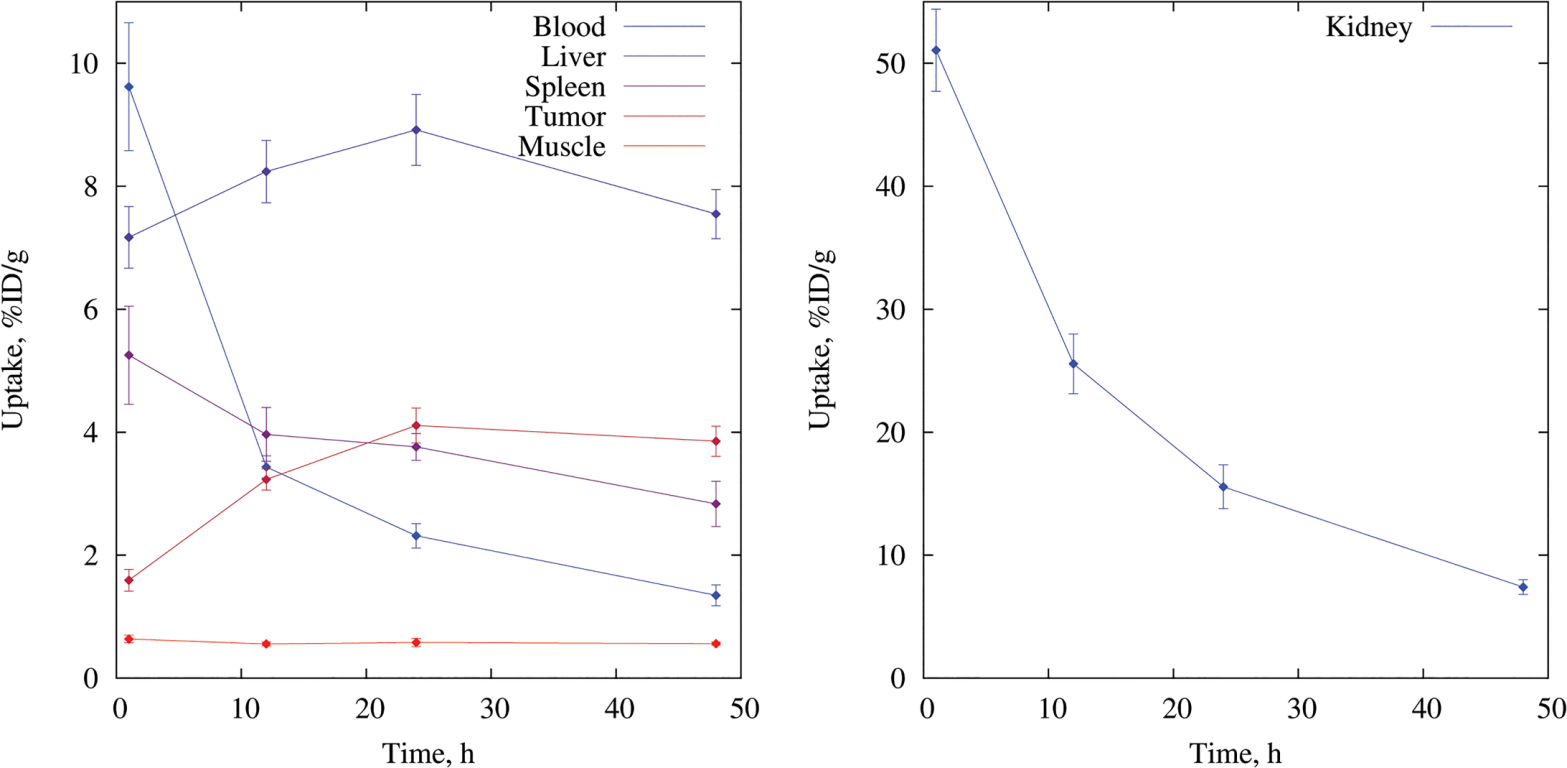
Biodistribution of nanodiscs from PET. The values were obtained from quantitative analysis of PET images extracted by region of interest (ROI) image analysis of tissues of interest, n = 6. Left: liver, spleen, tumor, muscle and left ventricle (blood), right: kidney. Each type of tissue was analyzed from PET/CT images obtained at 1, 12, 24 and 48 hours post-injection of ^64^Cu-nanodiscs by region of interest (ROI) image analysis. Results are presented as percentage injected dose per grams of tissue (%ID/g).

Analyzed PET images (Fig 5) showed that nanodiscs were cleared from the blood in a biphasic manner with an initial fast elimination consistent with the renal excretion followed by a much slower clearance enhancing the tumor accumulation beyond the 12 hour time-point [28]. The blood level dropped from 9.6% ± 1% ID/g 1 hour post injection (p.i.) to 1.3% ± 0.2% ID/g 48 hour p.i. and was significantly higher at 1 hour compared to the 12 hour, 24 hour and 48 hour time-points (p < 0.001), whereas no significant change was seen between the 12 hour, 24 hour and 48 hour observations. At 12 hour p.i. the blood and tumor levels were at similar levels. The tumor accumulation increased significantly from 1 hour to 12 hour p.i. (p < 0.001) and continued to increase up to 24 hour (12 hour vs 24 hour: p = 0.08), reaching 4%ID/g and remained steady until the end of the experiment at 48 hour p.i. (Fig 6). The steady increase in tumor accumulation together with the stabilized blood levels from the 12 hour time-point confirmed that the nanodiscs were circulating for a long time allowing extravasation through the leaky tumor vasculature.

**Fig 7:**
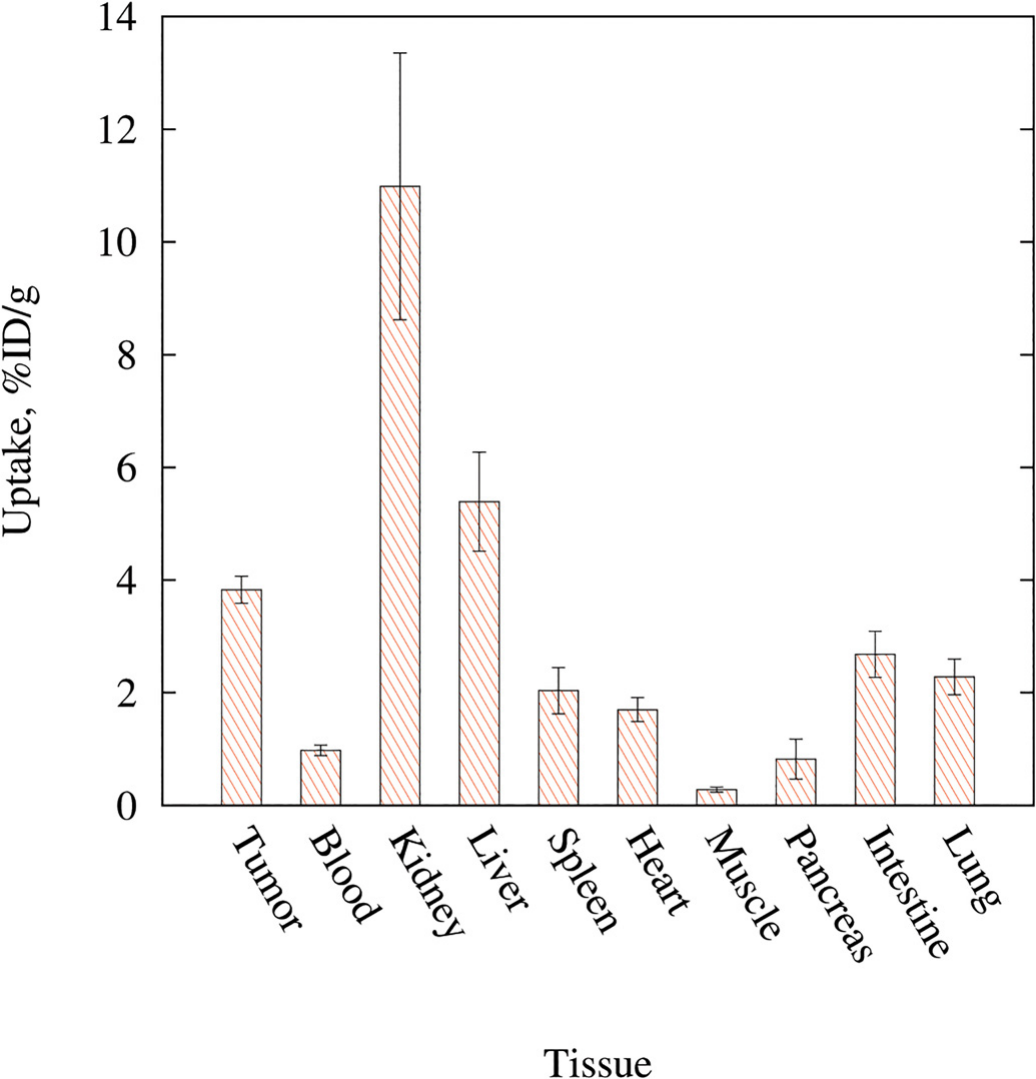
Biodistribution of nanodiscs from gamma detection. Assessed by gamma counting 48 hours post injection, n = 10. Dominating accumulation of ^64^Cu-nanodiscs was found in the kidneys. High accumulation was found in liver and tumor tissue as well.

The nanodiscs were primarily cleared through renal excretion, but the liver and intestinal uptake also reveals some excretion through the hepato-billiary route (Fig 7). Generally, nanoparticles and in particular nanoparticles without PEGylation suffer from recognition by the mononuclear phagocyte system, which results in accumulation in spleen and liver. Despite the nanodisc system being non-PEGylated, only a modest accumulation in the liver and the spleen was observed throughout the 48 hour study period. Accumulation in muscle tissue was low and remained steadily so throughout the experiment. For liver and muscle tissue, no significant alteration was observed throughout the 48 hour observation period, and we did not observe splenic accumulation over time. In fact the splenic uptake was significantly lower 48 hour p.i. compared to 1 hour p.i (p = 0.031).

Following the final PET/CT scan, mice were killed, and tissues of interest were collected for gamma-counter measurements. These results confirmed the results obtained by the quantitative analysis of PET data. Comparing the biodistribution in the different organs, we found that the uptake 48 hour p.i. was significantly higher in tumors compared to both blood (p < 0.001) and muscle (p < 0.001). The pancreatic uptake was low and only slightly higher than the muscle uptake (0.8 vs. 0.3%ID/g, p = 0.003), whereas some accumulation of the nanodiscs was observed in the lungs at similar levels as the spleen (2.3 vs 2.0%ID/g, p = 0.173). Uptake of high density lipoproteins (HDL) in lungs have previously been demonstrated [29] and given the resemblance of the nanodiscs to HDL, it is not surprising that we observe this lung accumulation.

There are several possible explanations for the observed renal clearance of the nanodiscs. Firstly, the ^64^Cu, observed in the kidneys, could be disassociated, if the radiochemical binding is unstable *in vivo*. However, as recently demonstrated, free ^64^CuCl_2_ accumulate in liver after the initial renal clearance to a higher degree than what we observed here [30] and ^64^Cu-DOTA would also be cleared faster than what we observe [23, 31, 32]. Secondly, disassembly of the nanodiscs in circulation would also prompt a renal clearance. HDL, which the nanodiscs resemble, are known to disassemble in vivo upon cholesterol transport. It is possible that what we see in the kidneys is ^64^Cu-labeled MSP disassociated from the phospholipid part of the nanodisc. However, we would have expected faster clearance of disassembled particles [33]. Therefore, we believe that the renal clearance rate is a matter of size and shape. The maximal dimension of the nanodiscs is approximately 130 Å, which is close to the cutoff for renal clearance of approximately 100 Å [34]. However, since the particles have the shape of approximately 50 Å thick ellipsoidal discs, it appears likely and consistent with the PET data that the nanodiscs can penetrate the kidney barriers enabling renal excretion of intact ^64^Cu-DOTA-nanodiscs.

It has been argued that ^64^Cu-DOTA is unstable *in vivo* prompting the evaluation of alternative chelates. However, chelates such as CB-TE2A do not seem to be superior to ^64^Cu-DOTA and requires much more harsh reaction conditions, which are unfavorable for protein labeling [31, 32]. We also believe that our in vivo data confirm sufficient in vivo stability due to the long blood circulation time and tumor accumulation up to 48h p.i.

The observed renal clearance is potentially of great importance for future clinical applications, as it represents a compromise between long blood circulation and ability for clearance from the body, thereby avoiding harmful accumulation in the body of potentially toxic products, if e.g. inorganic materials were to be carried with the nanodiscs[34]. However, the balance between renal and hepato-biliary clearance should be easily tunable through PEGylation of either the MSP or the lipids in the disc [35].

## Conclusion

With this study, we have provided new information on the biodistribution of nanodiscs, of central importance for future diagnostic and therapeutic applications and revealed a pattern which is very different from that observed from other well-known nanoparticles. Liposomes, which have been widely used as drug carriers [36], are found to accumulate to a high degree in liver and spleen apart from accumulation in tumor tissue [37]. Interestingly, this distribution pattern differs to a great extend from that observed for nanodiscs, which abundantly accumulates in the kidneys. Liver accumulation was observed for the nanodiscs as well but to a considerably lower degree. Hence, nanodiscs may be applied in cases, where a low liver accumulation is required. The low accumulation of nanodiscs in the spleen is also worth emphasizing, as this indicates a low immunogenicity of the nanodiscs evaluated. This is a general problem for several other nanoparticles and in particular for polymer based systems [38].

In conclusion, we have for the first time determined the biodistribution of nanodiscs *in vivo*. A simple and easy method for ^64^Cu radiolabelling of protein-based biomolecules that can be used for biodistribution studies by PET imaging was developed. We have characterized the DOTA-conjugated nanodiscs by SANS and SAXS and evaluated their biodistribution in tumor bearing mice in order to elucidate their potential for medical use. We find that the nanodisc particles penetrate very well into tumor tissue making them good candidates for drug delivery systems as well as imaging agents.

## Supporting Information

S1 File Fig A. DOTA-NHS ester conjugation of MSP. **Fig B.** Purification of DOTA modified nanodiscs.
(PDF)
