# Supplementary material for: PET/CT Based In Vivo Evaluation of 64Cu Labelled Nanodiscs in Tumor Bearing Mice
Source: PLoS One. 2015 Jul 1;10(7):e0129310. doi: 10.1371/journal.pone.0129310 (PMC4488450; doi:10.1371/journal.pone.0129310)
Supplement: S1 File — Fig B. Purification of DOTA modified nanodiscs. (PDF) [file pone.0129310.s001.pdf]

S1. PET/CT based *in vivo* evaluation of ^{64}Cu labelled nanodiscs in tumor bearing mice

The metal chelating reagent DOTA-NHS-ester (DOTA) was reacted onto lysine groups of the MSP, confirmed by MALDI TOF analysis (shown in Fig. A).

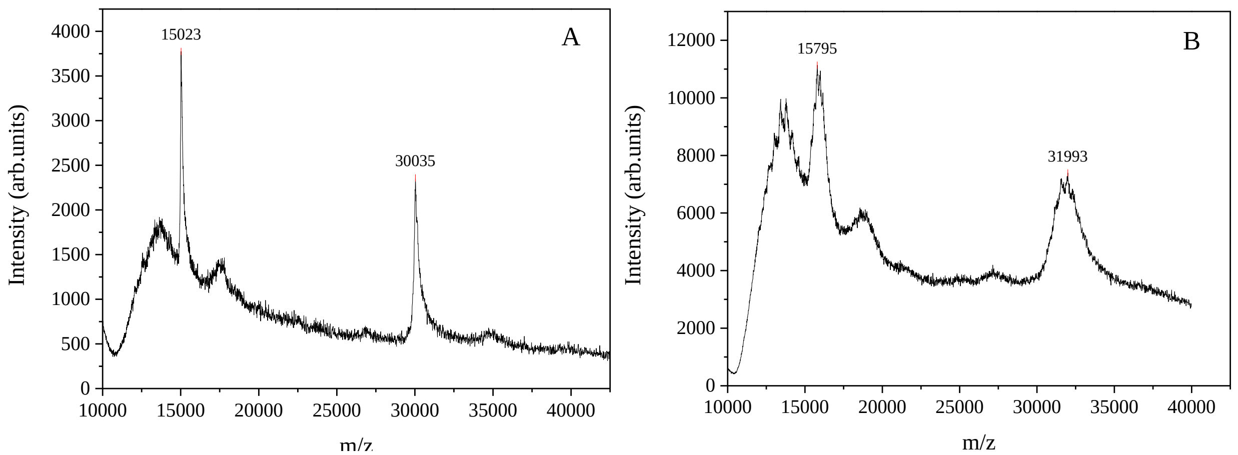

Fig. A. DOTA-NHS ester conjugation of MSP. Reaction confirmed by MALDI TOF. Spectrum of MSP before (A) and after (B) the reaction. An increase in mass to charge of approximately 2000 is observed corresponding to the addition of roughly 5 DOTA to 1 MSP.

DOTA modified nanodiscs exhibited the same reconstitution behavior as unmodified nanodiscs as judged from size exclusion chromatography (SEC) data from the purification process, and in both cases the well-defined discs were indicated (Fig. B).

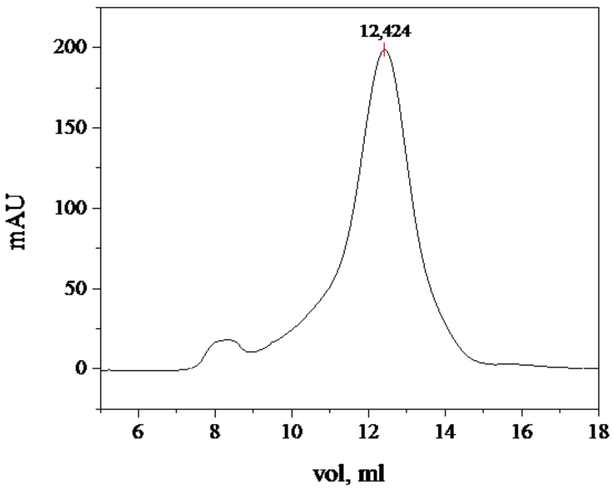

Fig. B. Purification of DOTA modified nanodiscs. Chromatogram obtained by size exclusion chromatography using a Superdex 200 (10/300) column detected by UV 280 nm absorption. ^{64}Cu-nanodiscs were collected from the column at an elution time of 12.4 ml.
